# Histone deacetylase activity mediates acquired resistance towards structurally diverse HSP90 inhibitors

**DOI:** 10.1002/1878-0261.12054

**Published:** 2017-04-11

**Authors:** Ryan C. Chai, Jessica L. Vieusseux, Benjamin J. Lang, Chau H. Nguyen, Michelle M. Kouspou, Kara L. Britt, John T. Price

**Affiliations:** ^1^ Department of Biochemistry and Molecular Biology Monash University Clayton Vic. Australia; ^2^ Bone Division Garvan Institute of Medical Research Darlinghurst NSW Australia; ^3^ Department of Radiation Oncology Centre for Life Sciences Beth Israel Deaconess Medical Center Harvard Medical School Boston MA USA; ^4^ College of Health and Biomedicine Victoria University Melbourne Vic. Australia; ^5^ Peter MacCallum Cancer Centre Melbourne Vic. Australia; ^6^ The Sir Peter MacCallum Department of Oncology University of Melbourne Vic. Australia; ^7^ Australian Institute for Musculoskeletal Science (AIMSS) Victoria University and University of Melbourne Vic. Australia; ^8^ Department of Medicine Melbourne Medical School‐Western Precinct The University of Melbourne St Albans Vic. Australia

**Keywords:** 17‐AAG, acquired resistance, cancer, histone deacetylases, HSP90

## Abstract

Heat shock protein 90 (HSP90) regulates multiple signalling pathways critical for tumour growth. As such, HSP90 inhibitors have been shown to act as effective anticancer agents in preclinical studies but, for a number of reasons, the same effect has not been observed in the clinical trials to date. One potential reason for this may be the presence of *de novo* or acquired resistance within the tumours. To investigate mechanisms of resistance, we generated resistant cell lines through gradual dose escalation of the HSP90 inhibitor 17‐allylamino‐17‐demethoxygeldanamycin (17‐AAG). The resultant resistant cell lines maintained their respective levels of resistance (7–240×) in the absence of 17‐AAG and were also cross‐resistant with other benzoquinone ansamycin HSP90 inhibitors. Expression of members of the histone deacetylase family (HDAC 1, 5, 6) was altered in the resistant cells. To determine whether HDAC activity contributed to resistance, pan‐HDAC inhibitors (TSA and LBH589) and the class II HDAC‐specific inhibitor SNDX275 were found to resensitize resistant cells towards 17‐AAG and 17‐dimethylaminoethylamino‐17‐demethoxygeldanamycin. Most significantly, resistant cells were also identified as cross‐resistant towards structurally distinct HSP90 inhibitors such as radicicol and the second‐generation HSP90 inhibitors CCT018159, VER50589 and AUY922. HDAC inhibition also resensitized resistant cells towards these classes of HSP90 inhibitors. In conclusion, we report that prolonged 17‐AAG treatment results in acquired resistance of cancer cells towards not just 17‐AAG but also to a spectrum of structurally distinct HSP90 inhibitors. This acquired resistance can be inhibited using clinically relevant HDAC inhibitors. This work supports the potential benefit of using HSP90 and HDAC inhibitors in combination within the clinical setting.

**Abbreviations**
17‐AAG: 17‐allylamino‐17‐demethoxygeldanamycin
17‐DMAG: 17‐dimethylaminoethylamino‐17‐demethoxygeldanamycin
5‐FU: 5‐fluorouracil
Ac‐H3: acetylated histone 3
BA: benzoquinone ansamycin
ER: endoplasmic reticulum
FBS: fetal bovine serum
GA: geldanamycin
HAT: histone acetyltransferase
HDAC: histone deacetylase
HSF1: heat shock factor 1
HSP90: heat shock protein 90
MDR: multiple drug resistance
NQO1: NAD(P)H: quinone oxidoreductase
P‐gp: p‐glycoprotein
RB: retinoblastoma protein
SRB: sulforhodamine B
STR: short tandem repeat
TCA: trichloroacetic acid
TSA: trichostatin A

## 1 Introduction

Heat shock protein 90 (HSP90) is a molecular chaperone required for the biogenesis, stabilization and folding of many cellular proteins under both physiological and pathophysiological conditions. With respect to cancer, many client proteins of HSP90 are overexpressed and/or mutated oncoproteins, essential for cancer cell survival, growth and invasive potential (da Rocha Dias *et al*., 2005; Sato *et al*., 2000; Xu *et al*., 2001). Consistent with this, high HSP90 expression is observed in the majority of cancers enabling them to maintain functionally active oncoproteins (Whitesell and Lindquist, 2005). Therefore, cancer cells are dependent on HSP90 for growth and survival, and thus, HSP90 has emerged as a major therapeutic target for many cancer types.

Central to its function, HSP90 contains an ATP‐binding/ATPase activity domain within its N‐terminal region (Jhaveri *et al*., 2012). Geldanamycin (GA), a member of the benzoquinone ansamycin (BA) family of antibiotics, binds to this site and inhibits HSP90 functionality resulting in the depletion of its oncogenic client proteins via the ubiquitin‐proteasome pathway (Trepel *et al*., 2010). Although GA was identified as an effective HSP90 inhibitor, its clinical development was limited due to its hepatotoxicity in preclinical models (Page *et al*., 1997). Thus, the generation of GA derivatives more appropriate for clinical development led to the identification of a number of compounds including 17‐allylamino‐17‐demethoxygeldanamycin (17‐AAG), which became the first HSP90 inhibitor to undergo clinical cancer trials, providing ‘proof‐of‐concept’ studies for the use of HSP90 inhibitors as anticancer agents (Jhaveri *et al*., 2012). Currently, a number of next‐generation structurally distinct HSP90 inhibitors, many generated by rational drug design methodologies, are in clinical evaluation, both as single agents and in combination regimes (Butler *et al*., 2015; Tatokoro *et al*., 2015; Wang *et al*., 2016; Yong *et al*., 2016).

To date, limited objective clinical outcomes have been reported in early‐phase clinical trials of HSP90 inhibitors in a variety of cancers (Butler *et al*., 2015; Heath *et al*., 2008; Ronnen *et al*., 2006; Solit *et al*., 2008). Preclinical and clinical data have highlighted a number of potential reasons for this including dose‐limiting toxicities such as ocular toxicity, the inability to stratify patients who would most likely benefit from HSP90 inhibitor treatment as well as the potential of the tumours to be resistant or acquire resistance towards HSP90 inhibitors limiting their overall efficacy (Butler *et al*., 2015). Pre‐existing (*de novo*) and acquired resistance towards anticancer drugs is a common feature in many cancers, thereby reducing their overall sensitivity towards first‐line chemotherapeutic treatments and targeted therapies. Although it was initially postulated that *de novo* and/or acquired resistance towards HSP90 inhibitors would be rare, due to HSP90′s central involvement in multiple concurrent pathways, it has emerged that mechanisms of resistance can exist. These studies have been primarily based upon the benzoquinone ansamycin (BA) family of antibiotics such as GA and 17‐AAG and has demonstrated that some key determinants of cellular resistance include high expression levels of client oncoproteins such as HER2 (Smith *et al*., 2001), the heat shock transcription factor 1 (HSF1) (Chen *et al*., 2013) and other heat shock proteins as well as HSP90 co‐chaperones (Guo *et al*., 2005; Holmes *et al*., 2008; McCollum *et al*., 2006). Cell cycle and apoptotic regulators, including the retinoblastoma protein (RB), p53, BAGs, and BAX, have also been shown to contribute to the sensitivity of cells towards HSP90 inhibitors (Maloney *et al*., 2003). Resistance towards the BA class of HSP90 inhibitors has also been linked to the multiple drug resistance (MDR) phenotype due to the up‐regulation of the drug efflux pump, p‐glycoprotein (P‐gp or MDR1), demonstrating that certain HSP90 inhibitors can be substrates for MDR1, although mutations are not required (Kim *et al*., 2015; Zhang *et al*., 2005). In addition, resistance towards the quinone‐containing BAs can be attributed to low levels of NAD(P)H: quinone oxidoreductase (NQO1), responsible for metabolizing drugs such as 17‐AAG, to their more potent semiquinone and hydroquinone forms, leading to increased HSP90 inhibition (George *et al*., 2005). Moreover, post‐translational modification of HSP90, such as acetylation, has been shown to be critical in maintaining normal chaperone function, in particular client protein and co‐chaperone binding (Kovacs *et al*., 2005; Scroggins *et al*., 2007).

In addition to *de novo* resistance, exposure of cancer cells to anticancer drugs can result in the acquisition of chemoresistance. This can occur due to induced genetic mutations as a result of drug pressure or the active selection and outgrowth of rare populations of cancer cells possessing a resistant genotype. Moreover, epigenetic changes can be a crucial driving force behind the acquisition of drug resistance. Indeed, studies of drug‐resistant cell lines have shown that multiple changes in histone acetylation and CpG island methylation are present and can be induced by drug treatment (Baker *et al*., 2005; Wei *et al*., 2003). These changes may not only generate cells that are drug resistant but may also confer growth and survival advantages allowing for a more aggressive tumour phenotype. As repeated administration of HSP90 inhibitors has been required for treatment efficacy, exemplified by early‐phase trials of 17‐AAG and second‐generation HSP90 inhibitors whereby weekly administration of drugs is carried out for up to 6 months (Dickson *et al*., 2013; Heath *et al*., 2008; Solit *et al*., 2008), it is imperative to evaluate whether chronic HSP90 inhibitor treatment results in acquired resistance and whether this can be suppressed. Previously, a lung cancer cell line with acquired resistance towards GA and 17‐AAG has been described with the induction of heat shock proteins being shown to have an important role in conferring resistance towards 17‐AAG (McCollum *et al*., 2008). Low NQO1 levels are also known to play a significant role in the underlying mechanism of acquired resistance towards 17‐AAG in glioblastoma and melanoma cell lines (Gaspar *et al*., 2009). These studies generated cell resistance by rapid dose escalation of GA or 17‐AAG, and although these models were highly resistant to other BA HSP90 inhibitors, they did not display any cross‐resistance to other structurally distinct classes of HSP90 inhibitor compounds (Gaspar *et al*., 2009; McCollum *et al*., 2006, 2008). Therefore, identifying mechanisms of resistance and defining ways to inhibit this, especially with respect to next‐generation HSP90 inhibitors that are currently in clinical trials, is of high importance.

In the current study, we have determined that gradual dose escalation of 17‐AAG in cancer cell lines leads to acquired resistance, not only towards 17‐AAG and other BAs, but most significantly towards the structurally distinct classes of next‐generation HSP90 inhibitors such as AUY922. More significantly, we determined that an important mechanism of resistance towards acquired resistance towards a wide spectrum of HSP90 inhibitors is through altered HDAC expression and that the use of clinically relevant HDAC inhibitors can reverse resistance towards both BA and next‐generation HSP90 inhibitors. These findings represent an important step towards identifying other clinically relevant therapies that can be provided in conjunction with HSP90 inhibitors to increase their overall efficacy and utility.

## 2 Materials and methods

### 2.1 Cell lines and cell culture

The human cancer cell line MDA‐MB‐435 was a kind gift from Janet Price (MD Anderson Cancer Centre, University of Texas, Houston, TX, USA). The mammary epithelial origin of the MDA‐MB‐435 cell line has been questioned in recent years and was recently reclassified as being melanocytic in origin rather than mammary (Rae *et al*., 2007; Ross *et al*., 2000) although it is reported to display strong phenotypic characteristics of a breast cancer cell line (Chambers, 2009; Sellappan *et al*., 2004). Despite this, the MDA‐MB‐435 cell line still represents a well‐characterized model of an advanced mesenchymal cancer cell phenotype. The human breast cancer cell line MDA‐MB‐231 was obtained from ATCC (Manassas, VA, USA). Both cell lines were maintained in Dulbecco's modified Eagle's medium (DMEM) (Life Technologies, Grand Island, NY, USA) containing 10% fetal bovine serum (FBS; Thermo Fisher Scientific, Rockford, IL, USA) and antibiotic/antimycotic (Life Technologies). All assays utilized this medium (DMEM/FBS). The consistency of cell genotypes and identities were confirmed by short tandem repeat (STR) profiling performed at Cell Bank Australia (Westmead, NSW, Australia). For clarity, the cell lines are referred to as MDA‐435 and MDA‐231, respectively, from here on.

### 2.2 Compounds

17‐Allylamino‐17‐demethoxygeldanamycin was obtained from Merck‐Calbiochem, Darmstadt, Germany; geldanamycin (GA), 17‐dimethylaminoethylamino‐17‐demethoxygeldanamycin (17‐DMAG), radicicol, LBH589 and SNDX275 were obtained from LC Labs (Woburn, MA, USA). Coumermycin A1, novobiocin and trichostatin A (TSA) were obtained from Sigma Aldrich (St. Louis, MO, USA). NVP‐AUY922, CCT018159 and VER50589 were obtained from Cayman Chemicals (Ann Arbor, MI, USA). The P‐gp inhibitor (verapamil) and the chemotherapeutic agents paclitaxel, doxorubicin and 5‐fluorouracil (5‐FU) were obtained from Merck‐Calbiochem.

### 2.3 Growth inhibition studies

Growth inhibition was determined using the sulforhodamine B (SRB) assay as described by Skehan *et al*. (1990). Briefly, 5 × 10^3^ cells were seeded into 96‐well plates in triplicates, allowed to attach overnight, after which time drug was added to the wells. After three days of exposure to the drug, cells were fixed with cold 50% trichloroacetic acid (Sigma Aldrich) for 1 h at 4 °C and stained with 0.4% SRB (Sigma Aldrich) in 1% acetic acid for 10 min at room temperature. Unbound SRB stain was then rinsed with 1% acetic acid after which the plates were left to air‐dry overnight. SRB stain was then solubilized in 150 μL of Tris/HCl (pH 10.5). Absorbance at 550 nm was measured using a Multiskan FC Absorbance plate reader (Thermo Fisher Scientific). The IC_50_ was calculated using GraphPad Prism (San Diego, CA, USA) as the drug concentration that inhibited cell growth by 50% compared to control cell growth.

### 2.4 Development of 17‐AAG acquired resistant cell lines

MDA‐435 and MDA‐231 cells were incubated in T75 flasks at 1 × IC_50_ concentrations of 0.05 μm and 1 μm 17‐AAG, respectively, as previously determined. Surviving MDA‐435 cells were allowed to grow to confluence after which they were passaged into another flask and the concentration of 17‐AAG was increased gradually from 0.1 to 2 μm over 10 incremental steps with appropriate passaging until cells maintained stable growth. Similarly, 17‐AAG concentration was increased gradually in the MDA‐231 cells from 1 to 6 μm. Untreated cells and cells treated with vehicle control were serially passaged along with 17‐AAG‐treated cells; no changes in phenotype or sensitivity to 17‐AAG were observed in the control and vehicle‐treated cells. After the cells were confirmed to be stably resistant to 17‐AAG by growth inhibition assays, cells were grown in 17‐AAG‐free DMEM/FBS. The consistency of cellular genotypes and identities were confirmed by short tandem repeat (STR) profiling performed at Cell Bank Australia (Westmead, NSW, Australia).

### 2.5 Western blot analysis

Western blot analysis was performed as described previously (Price *et al*., 2005). Briefly, cells were plated in six‐well plates, allowed to adhere overnight and then treated as described. Cells were rinsed twice with ice‐cold PBS, lysed and lifted from the plate by scraping in modified RIPA lysis buffer (50 mm Tris/HCl, 150 mm NaCl, 1% NP‐40, 0.25% sodium deoxycholate) containing protease and phosphatase inhibitors (Sigma Aldrich). After two‐minute sonication (4 × 30 s pulses) at 4 °C in a sonicating water bath, the lysate was clarified by centrifugation (14 500 ***g***) for 15 min at 4 °C. Total protein concentration was determined using bicinchoninic acid assay according to the manufacturer's protocol (Thermo Fisher Scientific). To prepare samples for SDS/PAGE, SDS sample buffer containing reducing agent (Life Technologies) was added to the protein samples which were then boiled for 5 min and centrifuged. Protein samples were then separated by one‐dimensional SDS/PAGE on a 4–12% gradient acrylamide gel (Life Technologies). The separated proteins were then transferred to polyvinylidene difluoride membranes (Merck‐Millipore, Bayswater, Vic., Australia), blocked with 3% skim milk and probed with primary antibodies at 4 °C overnight and then at room temperature with peroxidase‐conjugated secondary antibodies (Thermo Fisher Scientific) for 1 h. Proteins were visualized by an ECL detection system according to the manufacturer's protocol (Thermo Fisher Scientific). All antibodies were purchased from commercial sources. Antibodies that detected IGF‐1R (#3027), AKT (#9272), phosphorylated AKT (#4058), EGFR #2232), PDK (#3062), HDAC1 (#5356), HDAC3 (#5113), HDAC4 (#7628), HDAC5 (#2082), HDAC6 (#7558), and acetylated histone H3 (Lys27) (#4353) were purchased from Cell Signaling Technologies (Danvers, MA, USA). Antibodies that detected cyclin B1 (554176), CDK2 (610145) and pan‐actin (612656) were purchased from BD Pharmingen (CA, USA), and antibodies that detected HSP27 (ADI‐SPA‐803) and HSP70 (ADI‐SPA‐812) were purchased from Enzo Life Science (San Diego, CA, USA). Anti‐mouse IgG, anti‐rabbit IgG and anti‐goat IgG HRP‐conjugated secondary antibodies were obtained from Thermo Fisher Scientific.

### 2.6 Immunoprecipitation

Cell lysates were prepared as described previously and precleared with protein A/Sepharose beads (Merck‐Millipore) for 1 h at 4 °C after which the protein concentration was measured as described previously. Precleared lysate (0.5 mg) was added to 50 μL of antibody‐bead slurry and rotated overnight at 4 °C. Antibody complexes were washed thrice with lysis buffer and then resuspended in sample buffer. Acetylated lysine residues of a number of HSP90 isoforms were determined by SDS/PAGE and western blotting. Antibodies used included HSP90 (ADI‐SPA‐835), GRP94 (ADI‐SPA‐850) and TRAP1 (ADI‐SPA‐971) obtained from Enzo Life Science, and acetylated lysine (#9441) was obtained from Cell Signaling Technologies.

### 2.7 Semiquantitative qPCR and primers

Total RNA was isolated using the Qiagen RNeasy kit according to the manufacturer's instructions (Qiagen, Germantown, MD, USA). One to two micrograms of total RNA was used to synthesize cDNA using the superscript VILO cDNA synthesis kit according to the manufacturer's instructions (Life Technologies). The synthesized cDNA underwent PCR amplification using the Expand High Fidelity PCR System (Roche Applied Science, Indianapolis, IN, USA). The primers used were as follows: NQO1 (FWD): 5′‐GGGATCCACGGGGACATGAATG‐3′, NQO1 (REV): 5′‐ATTTGAATTCGGGCGTCTGCTG‐3′. Amplifications were performed with the following profile: 95 °C 3 min; 30 × (95 °C 30 s, 60 °C 30 s, 72 °C 30 s); 72 °C 7 min; ended at 25 °C. The PCR products were run on 2% agarose gel.

### 2.8 Statistical analysis

Data are presented as mean ± SD. All assays were analysed by the unpaired Student's *t*‐test using GraphPad Prism. Significance is represented as *P < 0.05, **P < 0.01 and ***P < 0.001.

## 3 Results

### 3.1 17‐AAG chronic exposure leads to cancer cell acquired resistance towards 17‐AAG and other benzoquinone ansamycin HSP90 inhibitors

**Figure 1:**
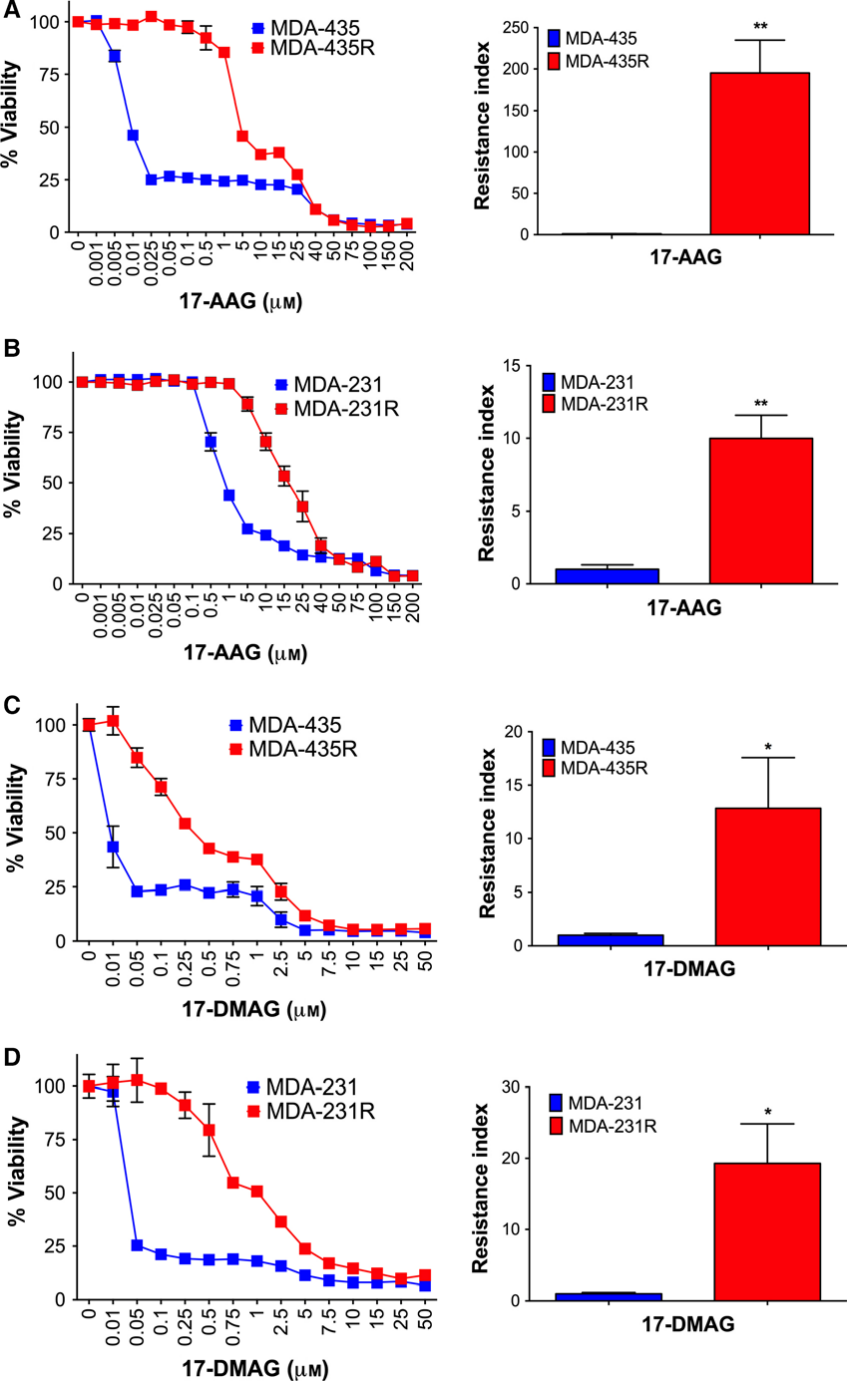
Acquired resistance of MDA‐435 and MDA‐231 cells towards 17‐AAG and 17‐DMAG. Dose–response curves and resistance index (RI = IC_50_ ratio relative to parental cell line) for MDA‐435‐ and MDA‐435R‐resistant cells treated for 3 days with 17‐AAG (A) and 17‐DMAG (C). MDA‐231‐ and MDA‐231R‐resistant cells treated for 3 days with 17‐AAG (B) and 17‐DMAG (D). *Growth curves*, representative of four independent experiments; *bars*, SD. *Columns*, mean of four independent experiments; *bars*, SD. *P < 0.05, **P < 0.01, ***P < 0.001 using Student's *t*‐test.

**Figure 2:**
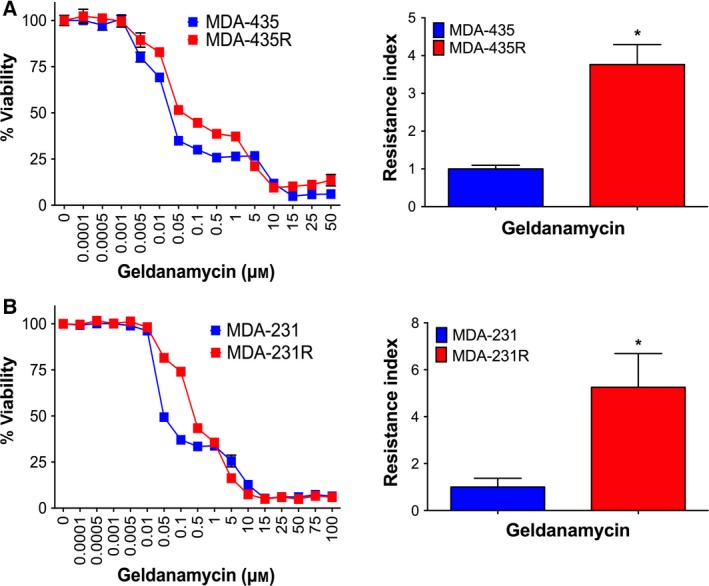
Cross‐resistance of MDA‐435R and MDA‐231R cells towards geldanamycin. Dose–response growth and resistance index of MDA‐435R (A) and MDA‐231R (B) treated with geldanamycin determined by SRB assay after 72 h. *Curves*, representative of three independent experiments; *bars*, SD. *Columns*, mean of at least two independent experiments; *bars*, SD. *P < 0.05, **P < 0.01 and ***P < 0.001 using Student's *t‐*test.

To investigate whether prolonged treatment with 17‐AAG would lead to an acquired resistance towards this agent, human cancer cell lines MDA‐435 and MDA‐231 were treated with increasing concentrations of 17‐AAG over a period of 20 and 15 weeks, respectively. SRB assays revealed that parental MDA‐435 cells were sensitive to 17‐AAG with an IC_50_ of 0.03 μm, while parental MDA‐231 cells were relatively resistant with an IC_50_ of 0.9 μm, respectively (Fig. 1A,B; Table 1). Cells that survived prolonged 17‐AAG exposure and became stably resistant *in vitro* to the highest concentrations of 17‐AAG were designated MDA‐435R and MDA‐231R, and these cells had IC_50_ concentrations of 7.12 and 10.35 μm, respectively (Fig. 1A,B; Table 1). Thus, MDA‐435R and MDA‐231R cells displayed high levels of 17‐AAG resistance with resistance indices (RI = IC_50_ resistant line/IC_50_ parental line) of 195 and 7.2, respectively. These 17‐AAG‐resistant cells also displayed significant resistance towards other structurally related HSP90 inhibitors such as other members of the BA family. For example, towards 17‐DMAG, the MDA‐435R and MDA‐231R had RIs of 12 and 24, respectively (Fig. 1C,D; Table 1). Moreover, the resistant cells showed a low but significant level of resistance towards GA with RIs of 3.8 and 5.3 for MDA‐435R and MDA‐231R, respectively (Fig. 2; Table 1). The IC_50_ values for all cell lines to 17‐AAG structurally related drugs are summarized in Table 1. These data demonstrate that chronic treatment with 17‐AAG leads to not only resistance towards 17‐AAG, but also the development of cross‐resistance towards structurally related BA HSP90 inhibitors in the cancer cell lines tested.

**Table 1:** Sensitivity of parental and resistant lines of MDA‐435 and MDA‐231 cancer cell lines to benzoquinone ansamycins (BAs) and structurally unrelated HSP90 inhibitors, as determined by IC_50_ values

	IC_50_ (μm)					
	MDA‐435	MDA‐435R	*P*‐value	MDA‐231	MDA‐231R	*P*‐value
17‐AAG	0.03 ± 0.01	7.12 ± 1.47	0.001***	0.9 ± 0.27	8.65 ± 1.38	0.009**
17‐DMAG	0.054 ± 0.009	0.66 ± 0.31	0.026*	0.07 ± 0.01	1.44 ± 0.42	0.005**
GA	0.032 ± 0.003	0.12 ± 0.02	0.001***	0.07 ± 0.03	0.37 ± 0.1	0.008**
Radicicol	0.22 ± 0.01	0.8 ± 0.16	0.011*	0.31 ± 0.08	0.83 ± 0.04	0.015*
CCT018159	8.02 ± 2.21	21.08 ± 2.36	0.0003***	9.08 ± 1.64	31.25 ± 3.25	0.0005***
VER50589	0.1 ± 0.02	0.34 ± 0.05	0.002**	0.15 ± 0.03	0.7 ± 0.21	0.012*
NVP‐AUY922	0.013 ± 0.003	0.021 ± 0.003	0.05*	0.028 ± 0.007	0.042 ± 0.003	0.026*
Novobiocin	293.23 ± 44.13	351.53 ± 37.4	0.962	353.73 ± 75.16	316.57 ± 40.75	0.456
Coumermycin A1	14.62 ± 3.04	14.49 ± 3.1	0.156	24.42 ± 9.07	24.5 ± 3.82	0.493

Values are mean ± SD of at least three independent experiments. *P‐*values were calculated using standard unpaired Student's *t*‐test of parental and resistant cell lines.

*P‐value < 0.05, **P‐value < 0.01, ***P‐value < 0.001.

**Figure 3:**
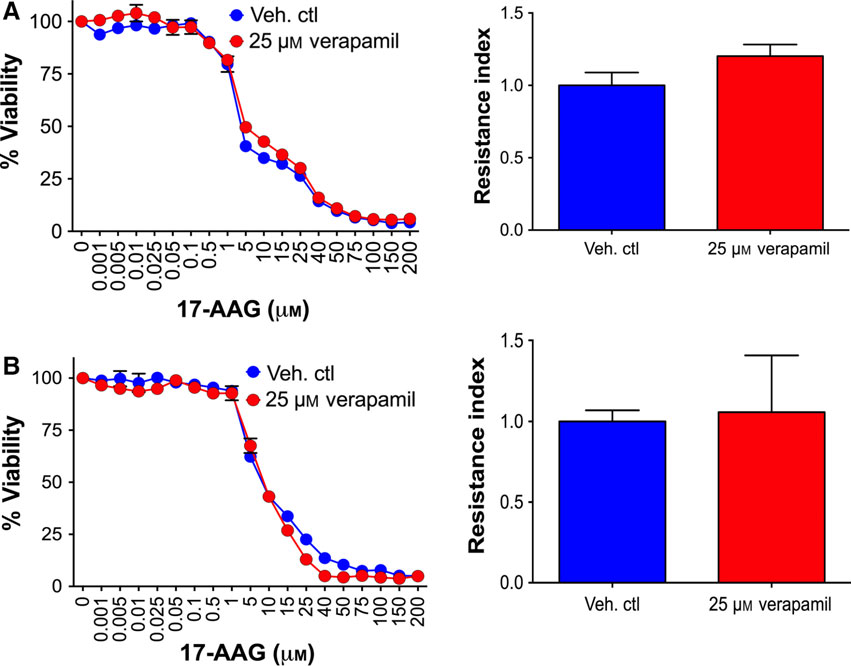
Inhibition of P‐gp by verapamil does not sensitize resistant cells towards 17‐AAG. Dose–response growth and resistance index of MDA‐435R (A) and MDA‐231R (B) treated with 17‐AAG alone or 17‐AAG with 25 mm of verapamil were determined by SRB assay after three days. *Curves*, representative of at least two independent experiments; *bars*, SD. *Columns*, mean of at least two independent experiments; *bars*, SD. *P < 0.05, **P < 0.01 and ***P < 0.001 using Student's *t*‐test.

To identify whether resistance was specific to BA HSP90 inhibitors and not due to the gain of a multidrug‐resistant phenotype through increased levels and/or activity of ATP‐dependent drug efflux pumps such as p‐glycoprotein 1 (P‐gp; MDR1; ABCB1), we determined IC_50_ values for chemotherapeutic agents known to be substrates of efflux pumps. Examination of cisplatin, paclitaxel, doxorubicin and 5‐FU in both the MDA‐435R and MDA‐231R demonstrated that there was no significant alteration in the response of the cells to these agents with the exception of MDA‐231R cells which were actually increased in their sensitivity towards doxorubicin (Table 2). Further support for this was our observation that the use of verapamil, a calcium channel blocker which inhibits P‐gp, failed to alter the resistance of MDA‐435R or MDA‐231R cells towards 17‐AAG (Fig. 3A,B, respectively). Therefore, taken together, these results indicated that the resistance was not mediated via a multidrug efflux pump mechanism.

**Table 2:** Sensitivity of parental and resistant lines of human cancer cells commonly used chemotherapeutic agents

	IC_50_ (μm)					
	MDA‐435	MDA‐435R	P‐value	MDA‐231	MDA‐231R	*P*‐value
Cisplatin	9.23 ± 0.84	12.26 ± 2.6	0.181	12.04 ± 1.94	6.89 ± 1.09	0.083
Paclitaxel	0.0027 ± 0.0006	0.003 ± 0.0006	0.568	0.0095 ± 0.004	0.0045 ± 0.0017	0.145
Doxorubicin	0.15 ± 0.03	0.17 ± 0.06	0.611	0.12 ± 0.02	0.07 ± 0.01	0.014*
5‐FU	5.98 ± 0.74	9.39 ± 2.55	0.09	17.59 ± 4.87	26 ± 4.20	0.086

Values are mean ± SD of at least three independent experiments. *P*‐values were derived from Student's *t*‐test of parental and resistant cell lines.

### 3.2 Sustained expression of HSP90 client proteins in MDA‐435R and MDA‐231R cells with 17AAG treatment

**Figure 4:**
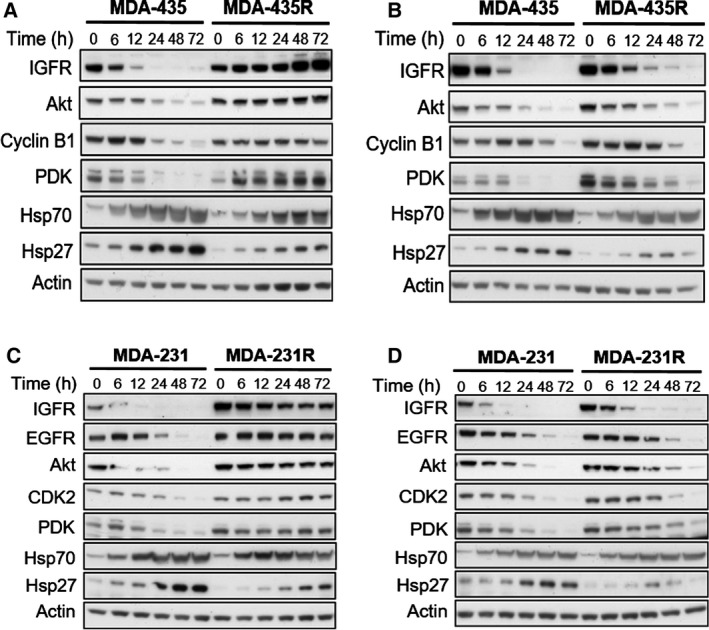
Molecular effects of HSP90 inhibition by 17‐AAG on HSP90 client proteins in parental and resistant cell lines. MDA‐435 parental and resistant MDA‐435R cells treated with 0.1 μm of 17‐AAG (~ 5 × 17‐AAG IC_50_ concentrations of the parental cell line) (A) and with 30 μm of 17‐AAG (~ 5 × 17‐AAG IC_50_ concentrations of the resistant cell line) (B). MDA‐231 parental and resistant MDA‐231R cells treated with 5.0 μm of 17‐AAG (~ 5 × 17‐AAG IC_50_ concentrations of the parental cell line) (C) and with 50 μm of 17‐AAG (~ 5 × 17‐AAG IC_50_ concentrations of the resistant cell line) (D). Total cell lysates were collected at the indicated time‐points and analysed by western blotting.

To investigate the molecular impact of acquired resistance towards 17‐AAG, we examined steady‐state levels of HSP90 client proteins as well as during 17‐AAG treatment of the parental and resistant cell lines. Parental MDA‐435 and resistant MDA‐435R cells were treated with 17‐AAG at 0.1 μm (~ 5 × IC_50_ of parental line) and 30 μm (~ 5 × IC_50_ of the resistant cell line). Treatment with 0.1 μm of 17‐AAG for 24 h resulted in the depletion of the HSP90 client proteins IGF‐1R, Akt, cyclin B1 and PDK in the parental MDA‐435 cells, which was not evident in the MDA‐435R cells (Fig. 4A). Induction of HSP70 and HSP27 was observed in both the parental and resistant cells; however, notably in the MDA‐435R cells, a lower degree of induction was observed (Fig. 4A). When treated with 30 μm of 17‐AAG (~ 5 × IC_50_ of MDA‐435R), depletion of the client proteins was observed in the MDA‐435R (Fig. 4B). The induction of HSP70 and HSP27 was evident in both cell lines treated at 30 μm of 17‐AAG, although the induction was lower in MDA‐435R when compared to parental cells (Fig. 4B). In a similar manner, parental MDA‐231 and resistant MDA‐231R lines treated with 17‐AAG at 5.0 μm (~ 5 × IC_50_ of the parental line) and 50 μm (~ 5 × IC_50_ of MDA‐231R) displayed similar results (Fig. 4C,D). Of note, the overall level and induction of HSP27 was markedly lower in the MDA‐231R cells as was observed in the MDA‐435R cells. These data demonstrate that HSP90 client protein levels are more stable in resistant cells upon 17‐AAG treatment as a consequence of acquired resistance. Furthermore, lower HSP levels, especially that of HSP27, may confer a survival advantage in the presence of 17‐AAG.

### 3.3 NQO1 is down‐regulated in 17‐AAG‐resistant cells

**Figure 5:**
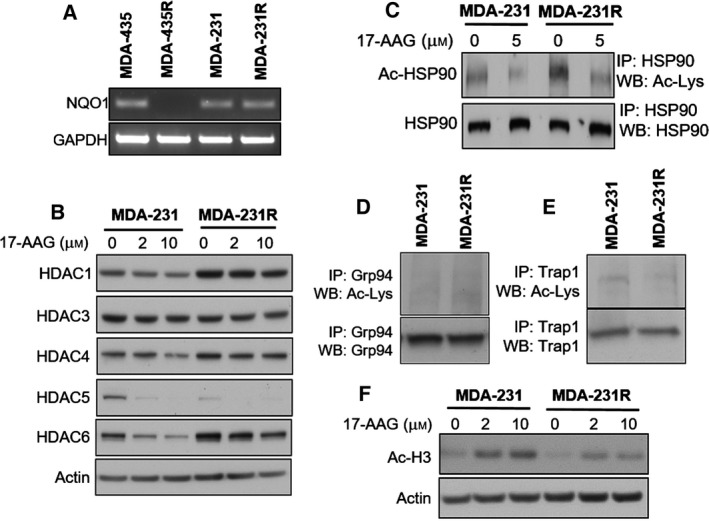
Altered NQO1 levels, HDAC family member expression and altered acetylation status in 17‐AAG‐resistant cell lines. Analysis of parental and resistant cell lines demonstrated altered expression levels of a number of molecules. Semiquantitative PCR demonstrated that the expression levels of NQO1 in resistant MDA‐435 cells were decreased when compared with parental cells, while no alteration was noted between MDA‐231 and MDA‐231R cell lines (A). Western blot analysis of parental and resistant MDA‐231 total cell lysates examining levels of HDAC family members in the presence and absence of 17‐AAG for a period of 24 h (B). Analysis of acetylated HSP90 by immunoprecipitation of HSP90 and western blot analysis with antiacetylated lysine antibody of total cell lysates of parental and resistant MDA‐231 cells treated with and without 17‐AAG demonstrated increased acetylated HSP90 (C). Analysis of acetylation of Grp94 (D) and Trap1 (E) by immunoprecipitation and western blot analysis of MDA‐231 and MDA‐231R total cell lysates demonstrated no alteration in acetylation status. Acetylated lysine residue was detected by western blotting. Western blot analysis of acetylated histone 3 in parental and resistant MDA‐231 cells treated with and without 17‐AAG demonstrated decreased nuclear acetylation (F).

In the light of the significant level of resistance to BAs, we examined the expression of NQO1 in the resistant cells as it has been previously shown to be a major mediator of resistance towards BA HSP90 inhibitors (Gaspar *et al*., 2009). NQO1 mRNA expression was significantly decreased in the MDA‐435R cells compared to the parental MDA‐435 cells as shown by semiquantitative RT‐PCR (Fig. 5A). However, interestingly, no difference in the mRNA expression levels of NQO1 was observed between MDA‐231 and MDA‐231R cells. These results indicate that decreased NQO1 expression may be a determinant of acquired resistance towards 17‐AAG and other BAs in the MDA‐435R cells as previously reported in other cancer cell lines; however, it does not appear to be a mode of resistance in the MDA‐231R cells, indicating other mechanisms of resistance exist in these cells.

### 3.4 Acquired resistance towards 17‐AAG alters HDAC levels and acetylation status of histone H3 and HSP90

During the examination of levels of HSP90 client proteins between the MDA‐231 parental and MDA‐231R cells, we identified that there were alterations in the steady‐state levels of HDAC family members. A more extensive analysis of HDAC expression demonstrated that HDAC 1 and 6 were expressed at higher levels in MDA‐231R cells, while HDAC 5 was significantly lower (Fig. 5B). Treatment of parental MDA‐231 cells with 17‐AAG decreased HDAC levels; however, as with other HSP90 client proteins, 17‐AAG had less impact upon HDAC levels in the MDA‐231R cells (Fig. 5B). The expression of HDAC 3 and 4 remained unchanged at both steady‐state levels or after 17‐AAG treatment in MDA‐231 and MDA‐231R cells (Fig. 5B). Interestingly, examination of the acetylation status of HSP90 demonstrated that despite increased levels of HDAC6 and HDAC1 in the MDA‐231R cells, HSP90 was actually increased in its acetylation status, while 17‐AAG treatment decreased the acetylation levels of HSP90 in both MDA‐231 and MDA‐231R cells (Fig. 5C). In other members of the HSP90 family, only limited levels of acetylation were identified and no alteration was detected in the endoplasmic reticulum (ER) (Fig. 5D) and mitochondria isoforms of HSP90, namely GRP94 (HSPC4) (Fig. 5D) and TRAP1 (HSPC4) (Fig. 5E), respectively. However, examination of acetylated histone 3 (Ac‐H3) demonstrated that decreased acetylation was evident in the MDA‐231R cells when compared with the MDA‐231 parental cells (Fig. 5F). Treatment of both parental and resistant cells with 17‐AAG increased H3 acetylation; however, there was a marked differential between the parental and resistant cell lines (Fig. 5F).

### 3.5 Inhibition of HDACs in MDA‐231R cells increases sensitivity towards HSP90 inhibitors

**Figure 6:**
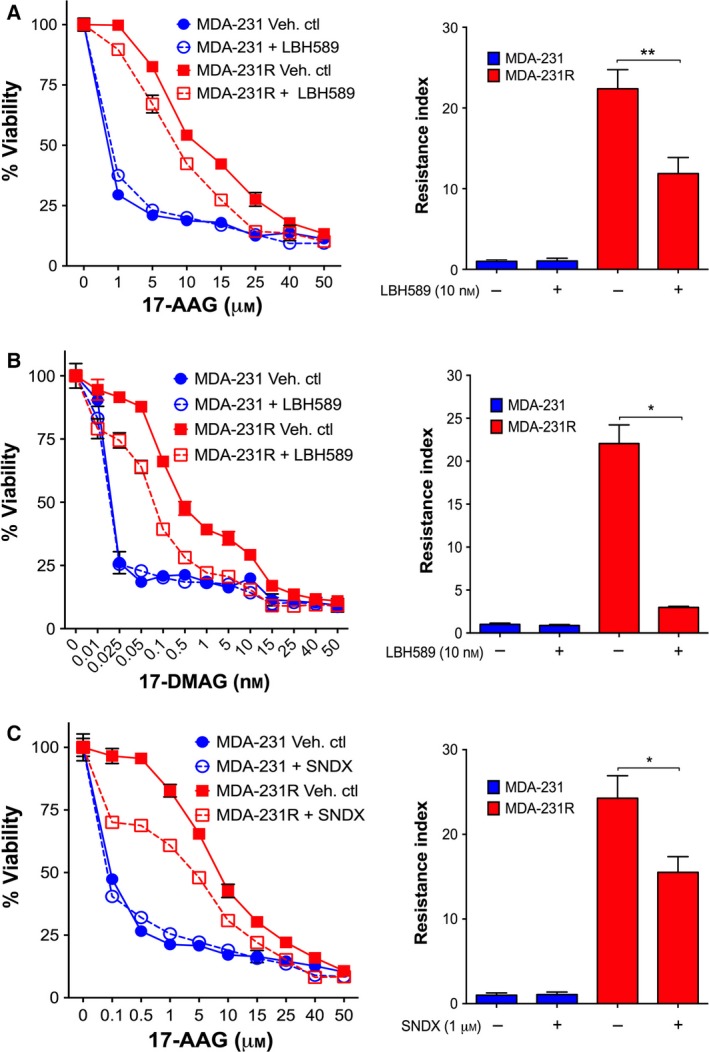
Inhibition of HDACs resensitizes MDA‐231R‐resistant cells towards 17‐AAG and 17‐DMAG. Dose response and resistance index (RI) of MDA‐231 and MDA‐231R cells treated with the pan‐HDAC inhibitor LBH589 (10 nm) in combination with 17‐AAG (A) or 17‐DMAG (B) for a period of 72 h. MDA‐231 and MDA‐231R cells treated with 17‐AAG (C) in combination with the more specific HDAC inhibitor SNDX275 (1 μm) that targets class I HDACs. *Growth curves*, representative of four independent experiments; *bars*, SD. *Columns*, mean of four independent experiments; *bars*, SD. *P < 0.05, **P < 0.001 using Student's *t*‐test.

In the light of the altered HDAC expression and acetylation status of the MDA‐231R cells, we investigated whether this may be a contributing factor to the acquired resistant phenotype. To test this, we examined whether pharmacological inhibition of HDAC family members would reverse HSP90 inhibitor resistance. We initially utilized the pan‐HDAC inhibitor LBH589 (panobinostat) and demonstrated that treatment of resistant MDA‐231 cells with LBH589 (10 nm) in combination with either 17‐AAG (Fig. 6A) or 17‐DMAG (Fig. 6B) resulted in a significant reversal of resistance in the MDA‐231R cells towards these BA HSP90 inhibitors as shown by decreased cell survival and RI (Fig. 6A,B). Interestingly, no significant change in the *de novo* resistance of the parental MDA‐231 cells was observed towards either 17‐AAG or 17‐DMAG when combined with LBH589.

In addition to using pan‐HDAC inhibitors, we also utilized SNDX‐275 (entinostat, formerly MS‐275), which targets class I HDACs, including HDAC1, up‐regulated in the MDA‐231R cells (Fig. 5B); however, it does not inhibit HDAC6 (Wardley *et al*., 2010; Younes *et al*., 2011). Consistent with class I HDAC activity being involved in acquired HSP90 inhibitor resistance, SNDX‐275 (1 μm) inhibited 17‐AAG resistance in the MDA‐231R cells (Fig. 6C). Notably, as with LBH589, the SNDX‐275 HDAC inhibitor did not impact on the sensitivity of parental MDA‐231 cells towards 17‐AAG (Fig. 6). The alteration in IC_50_ values of the MDA‐231R treated in combination with HDAC inhibitors is consistent with the up‐regulation and activity of HDACs, including the class I HDACs such as HDAC 1, contributing to acquired resistance towards BA HSP90 inhibitors (Table 3).

**Table 3:** Sensitivity of MDA‐231 parental and resistant lines towards benzoquinone ansamycins (BAs) and structurally unrelated HSP90 inhibitors and the impact of HDAC nhibtors as determined by IC _50_ values

	IC_50_ (μm)			
	MDA‐231	*P*‐value	MDA‐231R	*P*‐value
17‐AAG + Veh.	0.53 ± 0.04	0.8	10.58 ± 0.71	0.01**
17‐AAG + LBH589	0.57 ± 0.14		5.64 ± 0.9	
17‐DMAG + Veh.	0.03 ± 0.004	0.7	0.58 ± 0.06	0.013*
17‐DMAG + LBH589	0.02 ± 0.003		0.08 ± 0.003	
17‐AAG + Veh.	0.55 ± 0.06	0.9	11.64 ± 0.85	0.02*
17‐AAG + SNDX275	0.59 ± 0.08		7.62 ± 0.29	
Radicicol + Veh.	0.06 ± 0.01	0.4	0.51 ± 0.13	0.047*
Radicicol + LBH589	0.05 ± 0.0007		0.24 ± 0.03	
CCT018159 + Veh.	2.75 ± 0.46	0.7	20.92 ± 1	0.008**
CCT018159 + LBH589	2.52 ± 0.36		9.78 ± 0.02	

Values are mean ± SD of at least three independent experiments. *P*‐values were derived from Student's *t‐*test of parental and resistant cell lines.

### 3.6 17‐AAG‐resistant cells are cross‐resistant with next‐generation HSP90 inhibitors structurally unrelated to benzoquinone ansamycins

As decreased NQO1 expression is limited to conferring resistance towards BAs, we sought to investigate whether MDA‐435R and MDA‐231R displayed cross‐resistance with other HSP90 inhibitors that were structurally unrelated to GA, 17‐AAG, and 17‐DMAG, including next‐generation HSP90 inhibitors currently in clinical trial. Interestingly, both MDA‐435R and MDA‐231R cells were resistant to the HSP90 inhibitor radicicol, an antibiotic with a resorcinol scaffold and structurally unrelated to that of the BAs (Table 1). We also tested the sensitivity of the resistant cell lines to the resorcinylic compound, CCT018159, and the structure‐based designed pyrazole resorcinols VER50589 and AUY922, which have been shown to be more potent than GA derivatives in inhibiting HSP90 with their cellular potency being independent of NQO1 expression (Brough *et al*., 2007; Eccles *et al*., 2008; Sharp *et al*., 2007). Interestingly, both MDA‐435R and MDA‐231R cells showed significant resistance towards CCT018159 and VER50589 and to a lesser extent AUY922 (Table 1). This is the first report to demonstrate acquired resistance towards HSP90 inhibitors structurally unrelated to BAs in cancer cells. Of note, as MDA‐435R cells were also significantly resistant to the resorcinylic compounds, additional mechanisms of resistance other than reduced NQO1 levels are conferring resistance to non‐BA HSP90 inhibitors in the cells.

In contrast to resistance towards the N‐terminal ATPase HSP90 inhibitors, the resistant cell lines had no alteration in their response to known HSP90 inhibitors that are known to target the C terminus of HSP90, namely novobiocin and coumermycin A1 (Table 1).

### 3.7 Pharmacological Inhibition of HDACs reduces resistance towards HSP90 inhibitors structurally unrelated to benzoquinone ansamycins

**Figure 7:**
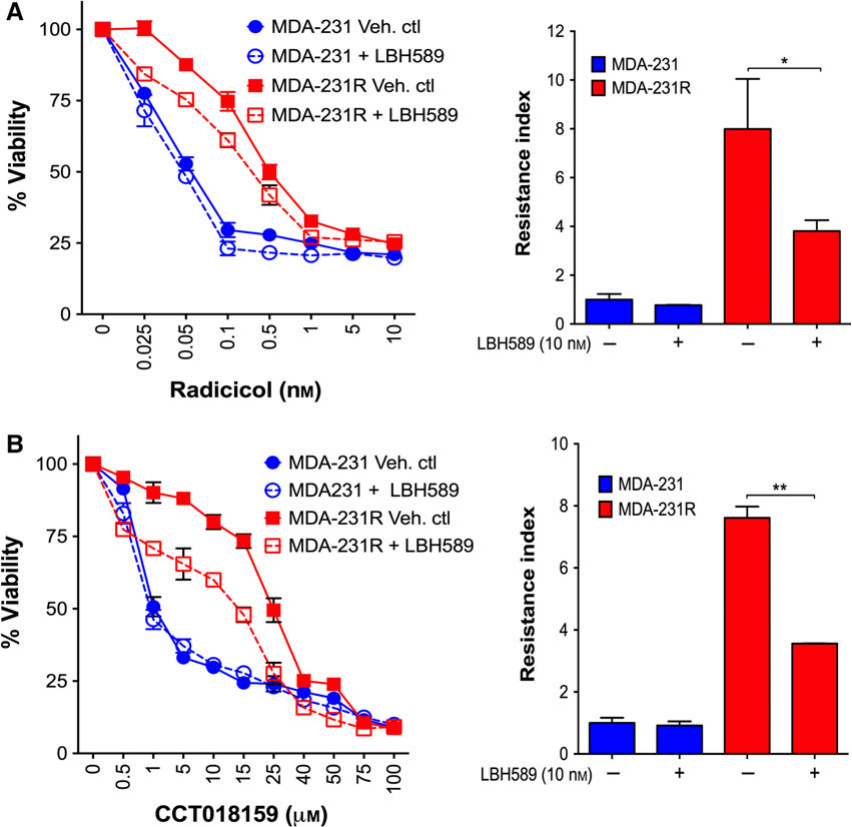
Inhibition of HDACs resensitizes MDA‐231R‐resistant cells towards structurally unrelated to the BAs and next‐generation HSP90 inhibitors. Dose response and resistance index of cells treated with radicicol (A) or with CCT018159 (B) in combination with LBH589 (10 nm) for a period of 72 h. *Growth curves*, representative of four independent experiments; *bars*, SD. *Columns*, mean of four independent experiments; *bars*, SD. *P < 0.05, **P < 0.01 using Student's *t*‐test.

To determine whether HDAC activity was not only involved in resistance towards BA HSP90 inhibitors but also towards the structurally distinct as well as next‐generation HSP90 inhibitors, we assessed whether resistance towards radicicol and CCT018159 could be inhibited through the cotreatment of MDA‐231R cells with the HDAC inhibitor LBH589. As with 17‐AAG and 17‐DMAG, pharmacological inhibition of HDAC family members in the MDA‐231R cells resulted in the reversal of resistance towards radicicol (Fig. 7A; Table 3) and CCT018159 (Fig. 7B; Table 3). Consistent with previous results, LBH589 did not increase the sensitivity of the MDA‐231 parental cells towards either HSP90 inhibitor (Fig. 7; Table 2). These results demonstrate for the first time that HDAC inhibition can reverse acquired resistance not only towards BA HSP90 inhibitors but also to structurally unrelated and next‐generation HSP90 inhibitors, thus indicating a role for HDACs in conferring acquired resistance towards diverse classes of HSP90 inhibitors.

## 4 Discussion

Therapeutically targeting HSP90 as an anticancer strategy has been a highly active area of research in recent years and has resulted in the generation of a number of clinically relevant HSP90 inhibitors, many of which have, or are progressing through clinical trials (Tatokoro *et al*., 2015; Whitesell *et al*., 2012). Despite optimism that HSP90 inhibitors would represent potent anticancer agents, many HSP90 inhibitor clinical trials have only shown modest activities or have failed to meet clinical endpoints (Cercek *et al*., 2014; Heath *et al*., 2008; Johnson *et al*., 2015; Ramalingam *et al*., 2015; Solit *et al*., 2008). A common problem associated with reduced treatment efficacy in tumours with the majority of anticancer treatments is the development of drug resistance. To date, few studies have systematically addressed this problem with respect to HSP90 inhibitors and none have examined acquired resistance towards next‐generation HSP90 inhibitors. Therefore, the current study aimed to generate models of acquired resistance towards the HSP90 inhibitor, 17‐AAG, identify the extent of cross‐resistance with conventional chemotherapeutics and next‐generation HSP90 inhibitors and seek to identify potential mechanism of resistance.

Our data demonstrated that chronic treatment of cancer cell lines with 17‐AAG led to acquired resistance towards not only 17‐AAG but also to a range of other structurally related HSP90 inhibitors. A highly significant finding of the study was that both MDA‐435R and MDA‐231R cell lines were also cross‐resistant to HSP90 inhibitors structurally unrelated and chemically distinct to the BAs, including radicicol and next‐generation HSP90 inhibitors CCT018159, VER50589 and AUY922. To our knowledge, this is the first report of cancer cells with acquired resistance that spans different classes of HSP90 inhibitors. However, it was noted that this cross‐resistance did not include C‐terminal HSP90 inhibitors such as novobiocin and coumermycin A1 as the resistant cells retained similar IC_50_ values towards these compounds.

Although this is the first study to demonstrate resistance to radicicol and next‐generation HSP90 inhibitors such as VER50589 and AUY‐922, acquired resistance towards 17‐AAG has been previously reported in lung, glioblastoma and melanoma cell lines (Gaspar *et al*., 2009; McCollum *et al*., 2008). As with the current study, these cells were found to be cross‐resistant with other members of the BAs, such as 17‐DMAG and GA; however, unlike our results, they were not identified as cross‐resistant to structurally unrelated HSP90 inhibitors.

Consistent with previous studies that decreased expression of the quinone‐reducing enzyme, NQO1, which metabolizes HSP90 inhibitors from the BA family to more potent hydroquinone forms, was a mode of resistance towards 17‐AAG and 17‐DMAG (Gaspar *et al*., 2009), we found NQO1 to be significantly reduced in MDA‐435R cells. However, NQO1 expression remained unchanged in the MDA‐231R cells, suggesting that there were other mechanisms of resistance towards BA members in these cells. Previously identified mechanisms of resistance such as increased P‐gp expression, an increased heat shock response and up‐regulation of HSP27 were not mechanisms by which HSP90 inhibitor resistance was achieved in the MDA‐231 cells (Gaspar *et al*., 2009; McCollum *et al*., 2006, 2008).

Identification of alterations in the levels of HDAC family members led us to hypothesize that acquired cell resistance towards different classes of N‐terminal HSP90 inhibitors may be due to altered acetylation levels of HSP90 itself or the impact of that HDACs have upon the epigenetic regulation of gene expression. Previous reports have shown that hyperacetylation of HSP90 lysine residues can affect co‐chaperone association, ATP binding and chaperone function, leading to reduced HSP90 function and increased client protein degradation (Rao *et al*., 2008). It has also been shown that hyperacetylation can result in increased binding of 17‐AAG to HSP90 resulting in increased inhibition and client protein degradation (Rao *et al*., 2008). The mainly cytosolic class IIb HDAC family member HDAC6 has been identified as one of the main deacetylases of HSP90 (Scroggins *et al*., 2007). In addition, class I HDAC family member, HDAC1, also has the potential to regulate HSP90 acetylation (Zhang *et al*., 2013). Broadly, it is thought that acetylation of HSP90 results in a lost or weakened interaction with its client proteins, leading to their instability and degradation (Kramer *et al*., 2014; Rao *et al*., 2008). Consistent with this, studies have shown that inhibition or knockdown of HDAC6 leads to increased acetylation of HSP90 and decreased activity (Kramer *et al*., 2014; Rao *et al*., 2008). However, this linear relationship between HSP90 hyperacetylation and decreased HSP90 activity and degradation of client proteins has been questioned, with recent evidence suggesting a more complex relationship between HSP90 hyperacetylation and activity such that in some cell types, hyperacetylation of HSP90 can lead to increased client protein levels (Kramer *et al*., 2014). Our finding that both HDAC6 and HDAC1 are increased at the protein level in the MDA‐231R cells would suggest that HSP90 should have lower steady‐state acetylation levels; however, counterintuitively, we found that HSP90 was more hyperacetylated in the MDA‐231R cells than in the MDA‐231 parental cells. Although the mechanism of this is not known, we speculate that this may be due to an overall net increase in histone acetyltransferase (HAT) activity over HDAC activity with respect to HSP90. Indeed, acetylation homeostasis in cells is a tightly regulated process with HDAC and HAT levels maintained in a fine balance (Peserico and Simone, 2011). HATs that target HSP90 remain largely unknown to date; however, the acetyltransferase p300 has been shown to be responsible for acetylating HSP90 at multiple sites (Yang *et al*., 2008). The possible role of p300 or other HATs in the regulation of HSP90 acetylation and resistance to HSP90 inhibitors requires further elucidation. No significant changes in the acetylation status of other HSP90 isoforms, namely GRP94 and TRAP1, localized to the ER and mitochondria, respectively, were evident in our studies.

Although there has been much emphasis within the field on the role of HDACs in the direct regulation of HSP90 activity, HDACs are intimately associated with transcriptional regulation through their ability to modify chromatin, alter transcription factor activity and impact upon transcriptional elongation (Greer *et al*., 2015; Wang *et al*., 2009). HDAC1 localization is predominantly nuclear and has been implicated in the regulation of gene expression (Zupkovitz *et al*., 2006), and while HDAC6 is known to be largely cytoplasmic, recent evidence indicates that it shuttles between the cytoplasm and nucleus and also plays a critical role in the regulation of gene expression (Greer *et al*., 2015; Liu *et al*., 2012; Wang *et al*., 2009). Consistent with increased HDAC activity at the nuclear level, acetylation of histone 3 in the resistant cells was decreased, while 17‐AAG treatment of both parental and resistant cells resulted in decreased HDAC expression and increased histone acetylation, although notably less in the resistant cells. Therefore, although previous studies showed that HDAC inhibition can sensitize leukaemia cells towards 17‐AAG through HSP90 hyperacetylation resulting in increased 17‐AAG binding and increased client protein degradation (Rao *et al*., 2008; Scroggins *et al*., 2007), we postulate that acquired HSP90 inhibitor resistance is mediated by an epigenetic mechanism. That is, through altered HDAC activity and the subsequent influence on the expression of a wide number of genes, HDACs are able to mediate a HSP90 inhibitor cell resistance phenotype. To investigate this further, we are currently examining gene expression profiles of resistant cells associated with resistance, which can be reversed with HDAC inhibition.

In summary, our results demonstrate for the first time that HDACs are involved in the underlying mechanism of acquired resistance towards multiple classes of HSP90 inhibitors, potentially through an epigenetic mechanism rather than through a direct impact upon HSP90 acetylation status; however, the latter cannot be fully discounted. These findings do, however, provide further support towards examining the use of HDAC inhibitors in combination with HSP90 inhibitors to overcome potential acquired resistance that may occur, thus increasing the therapeutic efficacy of current clinically relevant HSP90 inhibitors.

## Author contributions

RCC and JTP conceived and designed the study. RCC, JLV, BJL and CHN performed the experiments. RCC, MMK and KB analysed and interpreted the data. RCC and JTP wrote the manuscript.
